# Synthesis and characterization of novel 4-benzyloxyphenyl 4-[4-(n-dodecyloxy)benzoyloxy]benzoate liquid crystal

**DOI:** 10.3906/kim-2007-64

**Published:** 2021-02-17

**Authors:** Emine BALKANLI, Fatih ÇAKAR, Hale OCAK, Özlem CANKURTARAN, Belkız BİLGİN ERAN

**Affiliations:** 1 Department of Chemistry, Faculty of Arts and Science, Yıldız Technical University, İstanbul Turkey

**Keywords:** Inverse gas chromatography, liquid crystals, thermodynamics

## Abstract

Liquid crystal (LC) compound 4-Benzyloxyphenyl 4-[4-( *n* -dodecyloxy)benzoyloxy]benzoate (BDBB) was prepared and characterized. Inverse gas chromatography (IGC) was to be a beneficial analysis method for the research of thermodynamic characteristics of the new LC. Acetate and alcohol isomers were used to examine LC selectivity via the IGC technique at temperatures between 333.2 K and 483.2 K. The retention diagrams of *n* -heptane, *n* -octane, *n* -nonane, *n* -decane, undecane, dodecane, tridecane, *n* -butyl acetate, isobutyl acetate, ethyl acetate, *n* -propylbenzene, isopropylbenzene, ethylbenzene, chlorobenzene, and toluene on BDBB were plotted with temperatures of 483.2–493.2 K. Flory–Huggins interaction parameter and weight fraction activity coefficient at infinite dilution were researched for BDBB.

## 1. Introduction

Liquid crystals (LCs), which flow as liquid and are crystalline in their anisotropic physical properties, are of great interest in various industrial applications and basic science [1,2]. The design and synthesis of new LCs by changing terminals, linking, and lateral alkoxy groups are often carried out to understand the effect of molecular structure on LC properties and to make some of them usable for new technologies.

Calamitic molecules are a class of LCs widely used in applications due to their molecular architecture which enables the occurrence of the promising physicochemical properties by the modification of linking groups, terminal chains, and lateral substituents in the design. Phenyl benzoate derivatives are one of the most studied calamitic compounds. A chiral moiety, polar groups, or alkoxy chains localize at the terminal of the molecular structure, and traditional calamitic mesophases such as lamellar phases (smectic) and nematic phases occur thermotropically [3–8].

During the last 50 years, IGC has been a known physicochemical information source for assorted nonvolatile substances [9–13]. Application of the IGC method in the investigation of thermodynamic properties has recently become particularly attractive. Furthermore, IGC is a fast and definite technique for getting precious data about the main thermodynamic characteristics for a LC. Additionally, IGC is applied to evaluate interactions between LC and solvents because it is an extremely useful, available, inexpensive, and well-suited technique that supplies new insights for the physicochemical characterization of LCs. In IGC, the column is filled with LC; solvents are injected into the IGC column via an inert carrier gas with a constant flow at infinite dilution [14].

In this research, the synthesis, characterization, and mesomorphic properties of a phenyl benzoate-based calamitic LC 4-Benzyloxyphenyl 4-[4-( *n* -dodecyloxy) benzoyloxy] benzoate (BDBB) were presented, and the selectivity of BDBB was investigated by using acetates and alcohol isomers with a temperature range of 333.2–483.2 K. Thermodynamic interaction parameters were examined using *n* -heptane, *n* -octane, *n* -nonane, *n* -decane, undecane, dodecane, tridecane, *n* -butyl acetate, isobutyl acetate, ethyl acetate, *n* -propylbenzene, isopropylbenzene, ethylbenzene, chlorobenzene, and toluene with temperatures of 483.2–493.2 K by IGC. 

### 1.1. Thermodynamic characterization

The specific retention volume, Vgo is obtained using IGC results via the following [15–17]:

(1)Vg0=Q(tR-tA)J273.2/(Trw)

where Q is the carrier gas flow rate measured at the room temperature Tr, tR and tA are retention times of solvent and air, respectively, J is James–Martin gas compressibility; correction factor term is given by J = [3(p _i_ /p _0_ ) ^2^ - 1] / [2(p _i_ /p _0_ ) ^3^ - 1], where p _i_ is the pressure at the column inlet and p _0_ is the pressure at the column outlet, and w is the weight fraction of LC. 

Under IGC conditions, Flory–Huggins liquid crystal–solvent interaction parameters, χ _12_^∞^ are defined in Eq. (2): 

(2)χ12∞=Ln(273.2Rv2/p10Vg0V10)-(1-(V10/v2M2))-p10(B11-V10)/RT

where R is the universal gas constant; p_1_^0^, B_11_ and V_1_^0^ are, respectively, saturated vapor pressure, gaseous state second virial coefficient, and the molar volume of the solvent at temperature T; v_2_ is the specific volume of LC [18–21].

Ω_1_^∞^ is weight fraction activity coefficient of solvent at infinite dilution equal to:

(3)Ω1∞=Ln(273.2R/Vg0p10M1)-p10(B11-V10)/RT

where M_1_ is the molecular weight of the solvent.

The partial molar heat of mixing, ΔH_1_^∞^, is calculated by the following relationship:

(4)ΔH1∞=R[δ(LnΩ1∞)/1/T)]

The partial molar heat of sorption, ΔHS, of the solvent sorbed by the solute is determined by the following equation:

(5)ΔHs=-R[δ(LnVg0)/δ(1/T)]

The molar heat of vaporization, ΔHV , of the solvent can be described by:

(6)ΔHV=ΔH1∞-ΔHS

## 2. Experimental

### 2.1. Materials and methods

The retention times of the solvents were examined with a Hewlett-Packard 6890 N gas chromatograph equipped with a thermal conductivity detector (Hewlett-Packard, Palo Alto, CA, USA). The stainless steel column (3.2 mm i.d. × 1 m) was purchased from Alltech Associates, Inc. (Chicago, IL, USA). Chloroform was slowly evaporated, with the support material stirred into the LC solution; thus, LC was coated onto the Chromosorb-W (AW-DMCS-treated, 80/100 mesh). A 1-µL Hamilton syringe was used to inject 0.1 µL of each solvent into the column for infinite dilution. The column was conditioned overnight in a stream of helium (He) at 373.2 K. The loading percentage of the BDBB on the support was determined as 13.15% by calcination. During the experiments, He which was kept at a constant flow rate of 5 cm^3^/min was used as the carrier gas. Abbreviation, source, mass fraction purity, and CAS registry number of the chemicals are given in Table 1.

**Table 1:** Abbreviation, source, mass fraction purity, and CAS registry number of the chemicals.

Chemical name	Abbreviation	Source	Purity	CAS number
n-heptane	Hp	Merck	≥ 0.990	142-82-5
n-octane	O	Merck	≥ 0.990	111-65-9
n-nonane	N	Merck	≥ 0.990	111-84-2
n-decane	D	Merck	≥ 0.995	124-18-5
undecane	UD	Merck	≥ 0.990	1120-21-4
dodecane	DD	Merck	≥ 0.990	112-40-3
tridecane	TD	Merck	≥ 0.993	629-50-5
n-butyl acetate	nBAc	Merck	≥ 0.995	123-86-4
isobutyl acetate	iBAc	Merck	≥ 0.980	110-19-0
tert-butyl acetate	tBAc	Merck	≥ 0.990	540-88-5
ethyl acetate	EAc	Merck	≥ 0.995	141-78-6
n-butyl alcohol	nBAl	Merck	≥ 0.995	71-36-3
isobutyl alcohol	iBAl	Merck	≥ 0.990	78-83-1
tert-butyl alcohol	tBAl	Merck	≥ 0.995	75-65-0
n-propylbenzene	nPB	Merck	≥ 0.999	103-65-1
isopropylbenzene	iPB	Merck	≥ 0.990	98-82-8
ethylbenzene	EB	Merck	≥ 0.990	100-41-4
chlorobenzene	ClB	Merck	≥ 0.990	108-90-7
toluene	T	Merck	≥ 0.999	108-88-3
Ethyl 4-hydroxybenzoate	-	Merck	0.980-1.020	120-47-8
1-bromododecane	-	Sigma Aldrich	0.970	112-29-8
potasium carbonate	-	Merck	≥ 0.990	584-08-7
sodium hydroxide	-	Merck	≥ 0.970	1310-73-2
sodium chlorite	-	Fluka	≥ 0.990	7647-14-5
sodium dihydrogen phosphate monohydrate	-	Merck	0.990-1.020	10049-21-5
resorcinol	-	Merck	≥ 0.990	108-46-3
4-(Dimethylamino)pyridine	-	Merck	≥ 0.990	1122-58-3
4-benzyloxyphenol	-	ABCR	0.980	103-16-2
di-phosphorus pentoxide	-	Merck	≥ 0.970	1314-56-3
2-Butanone	-	Merck	≥ 0.990	78-93-3
N,N’-dicyclohexylcarbodiimide	-	Merck	≥ 0.990	538-75-0
Chromosorb W 80-100 mesh	-	Merck	-	68855-54-9

Anhydrous solvent dichloromethane was dried over diphosphorus pentoxide. Analytical thin-layer chromatography (TLC) was carried out on aluminum plates coated with silica gel 60 F254 for intermediates and BDBB LC. Silica gel 60 (pore size 60 Å, 230–400 mesh particle size; Merck, Kenilworth, NJ, USA) was used when performing column chromatography.

The new liquid crystalline compound BDBB was characterized as ^1^H-NMR and ^13^C-NMR (Bruker Avance III 500 spectrometer, in CDCl_3_ solution, with tetramethylsilane as internal standard; Bruker, Billerica, MA, USA).

A Mettler FP-82 HT hot stage and control unit (Mettler, Columbus, OH, USA), in conjunction with a Leica DM2700P polarizing microscope (PM) (Leica, Wetzlar, Germany), were used to examine the mesomorphic properties of LC (BDBB). Differential scanning calorimetry (DSC)-thermograms of BDBB were recorded on a PerkinElmer DSC-6 (PerkinElmer, Akron, OH, USA); heating and cooling rate: 10 °C min^-1^ in a nitrogen atmosphere.

### 2.2. Synthesis and characterization of BDBB

**Figure 1:**
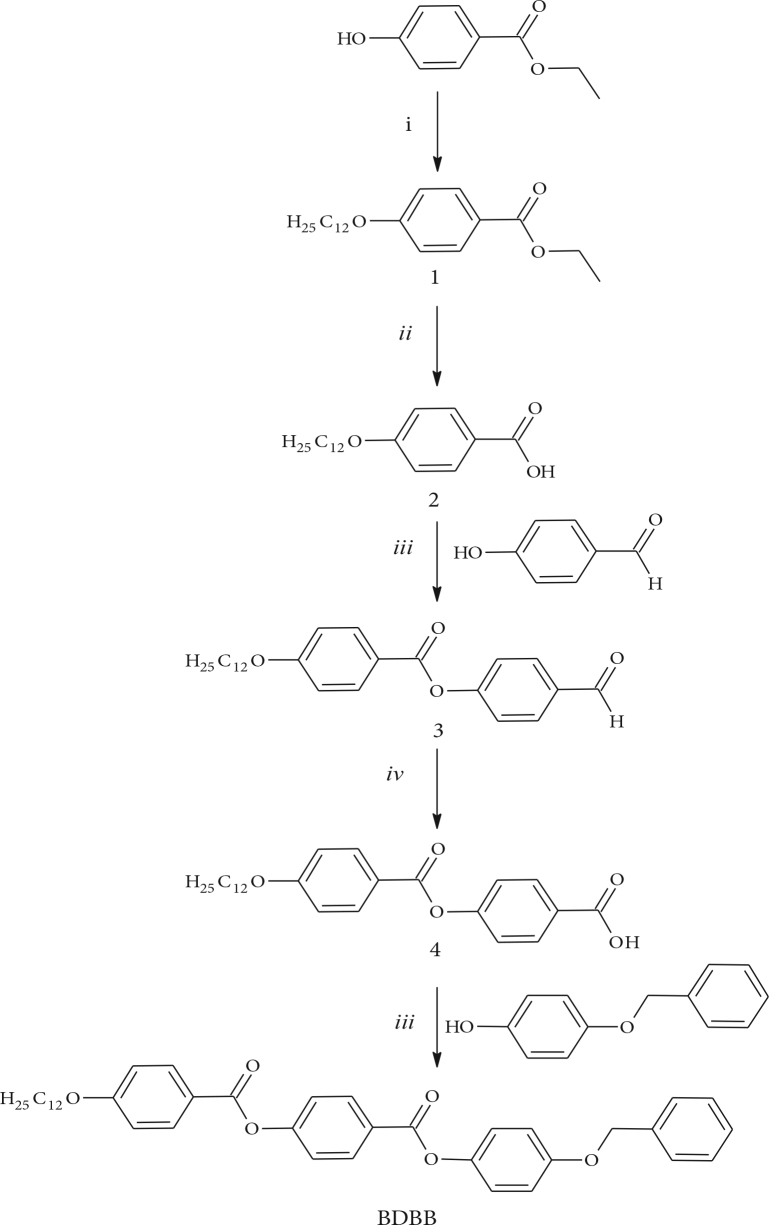
Synthesis of 4-Benzyloxyphenyl 4-[4-(n-dodecyloxy)benzoyloxy]benzoate (BDBB), Reagents and conditions: (i) C_12_H_25_Br, K_2_CO_3_, 2-butanone, reflux, 24 h; (ii) 10 N aqueous solution of NaOH, ethanol, reflux, 12 h; (iii) DCC, DMAP, CH_2_Cl_2_, r.t., 24h; (iv) NaClO_2_, NaH_2_PO_4_.H_2_O, tert-butyl alcohol, 24 h.

The synthesis of a new phenyl-benzoate–based calamitic molecule consisting of three aromatic rings with connected ester linking units 4-Benzyloxyphenyl 4-[4-( *n* -dodecyloxy)benzoyloxy]benzoate (BDBB) is shown in Figure 1. 

In the synthesis of BDBB, 4-(4- *n* -dodecyloxybenzoyloxy)benzoic acid (4) [22–24] was prepared starting with 1-bromododecane by using the procedure reported in the literature [25]. Commercially available ethyl 4-hydroxybenzoate was alkylated with 1-bromododecane, followed by hydrolysis of the ethyl group using 10 N sodium hydroxide solution in ethanol. Esterification of the obtained 4-( *n* -dodecyloxy)benzoic acid (2) with 4-hydroxybenzaldehyde using *N,N’* -dicyclohexylcarbodiimide (DCC) and 4-(dimethylamino)pyridine (DMAP) [26], followed by NaClO_2_ oxidation [27], led to 4-(4- *n* -dodecyloxybenzoyloxy)benzoic acid (4). Spectroscopic data of compound 4 is given in Muhammad et al. [28]. In the final step, 4-benzyloxyphenol was esterified by treatment with *n* -dodecyloxy–substituted benzoyloxybenzoic acid using *N,N’* -dicyclohexylcarbodiimide (DCC) and 4-(dimethylamino)pyridine (DMAP) as catalyst in dry CH_2_Cl_2_. The target benzoate-based calamitic compound BDBB was purified by column chromatography on silica gel using chloroform as eluent. 

The characterization of intermediate compounds and target compound BDBB is based on ^1^H, ^13^C-NMR (Bruker Avance III 500 spectrometer, in CDCl_3_ solution, with tetramethylsilane as internal standard). The proposed structure is in full agreement with the spectroscopic data (see Supporting Information).

### 2.3. The procedure for the synthesis of new benzoate-based calamitic molecule (BDBB)

In a 250-mL round-bottomed flask, 3.0 mmol of 4-(4- *n* -dodecyloxybenzoyloxy)benzoic acid (4), 3.3 mmol of 4-benzyloxyphenol, 4.8 mmol of *N,N’* -dicyclohexylcarbodiimide (DCC), and 0.3 mmol of 4-(dimethylamino)pyridine (DMAP) as catalyst were dissolved in 70 mL of dry dichloromethane and stirred at room temperature under an argon atmosphere for 24 h. The reaction was monitored by TLC (CHCl_3_). The resulting mixture was filtered on silica gel and washed with CH_2_Cl_2_. After removing the volatile components in vacuo, the crude product was purified by column chromatography on silica gel, eluting with CHCl_3_. 

**Yield:** 76% (1.38 g); colorless crystals. ^1^H–NMR (500 MHz, CDCl_3_): δ (ppm) = 8.34 (d, J ≈ 8.9 Hz; 2 Ar–H), 8.22 (d, J ≈ 8.9 Hz; 2 Ar–H), 7.53––7.40 (m, 7 Ar–H), 7.21 (d, J ≈ 8.9 Hz; 2 Ar–H), 7.09 (d, J ≈ 8.9 Hz; 2 Ar–H), 7.06 (d, J ≈ 8.9 Hz; 2 Ar–H), 5.15 (s, 2H, OCH2Bn), 4.12 (t, J ≈ 6.5 Hz; 2H, OCH2), 1.92––1.87 (m, 2H, OCH2CH2), 1.63––1.34 (m, 18H, 9 CH2), 0.95 (t, J ≈ 7.1 Hz; 3H, CH3). 13C–NMR (125 MHz, CDCl_3_): δ (ppm) = 164.88, 164.42, (COO), 163.88, 156.62, 155.36, 144.61, 128.10, 127.03, 121.00, (Ar–C), 136.88, 132.48, 131.82, 128.69, 127.55, 122.54, 122.12, 115.61, 114.47 (Ar–CH), 70.51 (OCH2Bn), 68.45 (OCH2), 31.98, 29.72, 29.70, 29.65, 29.62, 29.41, 29.14, 26.03, 22.75, (CH2), 14.19 (CH3).

## 3. Results

### 3.1. Liquid crystalline properties

PM and DSC were used to examine the mesomorphic characteristics of the new phenyl-benzoate–based calamitic molecule BDBB. The transition temperatures, corresponding enthalpy values, and mesophase type analyzed for BDBB are shown in Table 2. The transition temperatures are expressed in Kelvin units.

**Table 2:** The chemical structure, mesophase type, and phase transition temperatures of the BDBB.

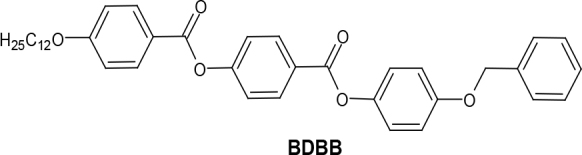	
Compound	T (K) (ΔH kJ/mol)
BDBBa	Heating: Cr 391.18 [45.82] SmC 432.15b N 467.82 [1.31] Iso
	Cooling: Iso 466.32 [-1.75] N 432.15b SmC 364.71 [-44.12] Cr

^a^Temperatures were expressed in Kelvin unit by adding 273.15 to Celsius degree. Perkin-Elmer DSC-6: enthalpy values were presented in brackets, heating, and cooling scans at a rate of 10 °C min^-1^. Abbrevations: Cr = crystalline, SmC = tilted smectic phase, N = nematic phase, Iso = isotropic phase. ^b^Determined by polarizing microscopy.

**Figure 2:**
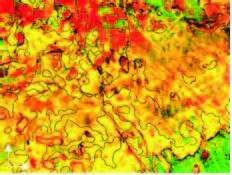

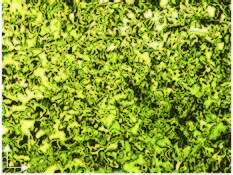

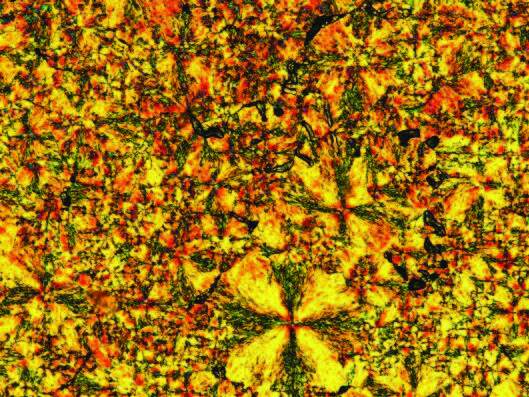
Optical textures of N and SmC mesophase of BDBB as observed between crossed polarizers (indicated by arrows) in nontreated microscopic glass slides on cooling (a) texture of N phase at T = 180.0 °C (b) Schlieren texture of SmC phase at T=145.0 °C (c) Crystalline state at T = 89.0 °C.

**Figure 3:**
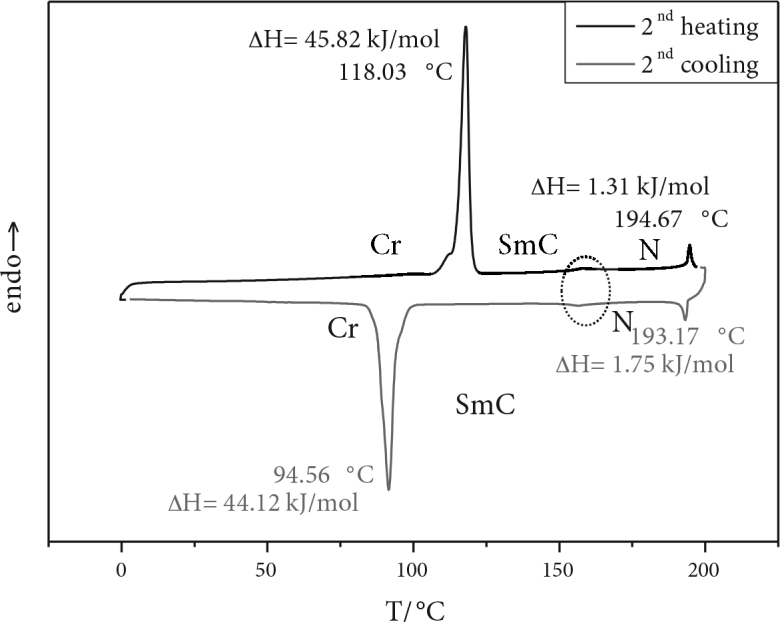
DSC thermograms of BDBB on 2nd heating and cooling (10 °C min^-1^).

The new calamitic LC compound BDBB shows an enantiotropic nematic (N) and tilted smectic (SmC) phase under polarizing microscope. The differential thermograms of BDBB show 2 endotherms for a phase transition of crystal (Cr)-smectic C (SmC) and nematic (N)-isotropic phase (Iso). On cooling from the isotropic phase, the same behavior of reverse transitions was observed. Additionally, a very broad transition peak corresponding to the SmC–N transition and vice versa appeared faintly on heating and cooling thermograms. However, the temperature of the SmC–N transition given in Table 2 was obtained by PM due to undetectable enthalpy changes by DSC. Typical textures of N and SmC mesophases observed for compound BDBB are shown in Figure 2, and a DSC thermogram is shown in Figure 3.

### 3.2. Thermodynamic interactions of BDBB

**Figure 4:**
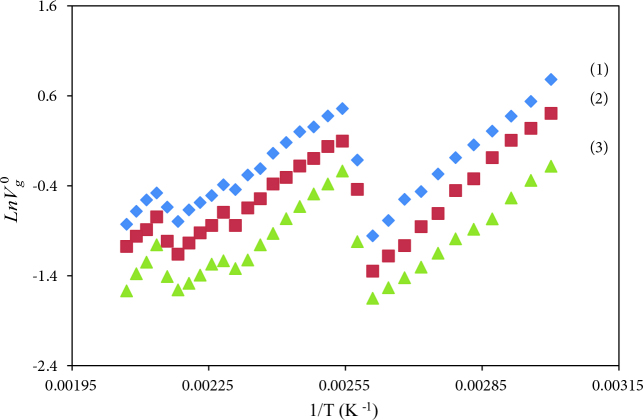
The specific retention volume diagrams, V_g_^0^, of nBAc (1), iBAc (2) and tBAc (3) on BDBB.

**Figure 5:**
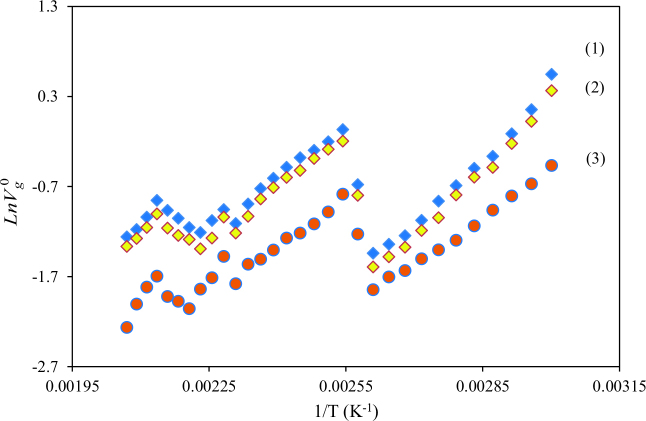
The specific retention volume diagrams, V_g_^0^, of nBAl (1), iBAl (2) and tBAl (3) on BDBB.

A description of the physicochemical or thermodynamic characteristics of an LC via IGC can be made by V_g_^o^, specific retention volume. IGC results include the V_g_^o^ of the isomeric solvents such as nBAc, iBAc, tBAc, nBAl, iBAl, and tBAl on BDBB with temperatures between 333.2 K and 483.2 K. The retention diagrams in Figures 4 and 5 were obtained from the plot of LnV_g_^o^ determined by Eq. (1) versus temperature. The percentage error in V_g_^o^ was calculated as less than ±0.4 by using 5 successive measurements of each datum.

With respect to the retention diagrams in Figure 4 and 5, Cr–SmC, SmC–N, and N–Iso transitions for BDBB occurred at 393.2 K, 438.2 K, and 468.2 K, respectively, as the maximum points point out. The Cr–SmC, SmC–N, and N–Iso transition temperatures tested via IGC analysis are in good accordance with the ones tested via DSC. According to Figures 4 and 5, convincing results were obtained in the separation of isomers between 333.2 K and 483.2 K in this study.

Thermodynamic measurements of BDBB in liquid state can be calculated at temperatures where thermodynamic equilibrium occurs. The stationary phase stays in the same thermodynamic state and linearity occurs. It can be seen in Figures 4 and 5 that the thermodynamic equilibrium region was between 483.2 K and 493.2 K. Hence the thermodynamic properties of BDBB were examined at these temperatures.

**Figure 6:**
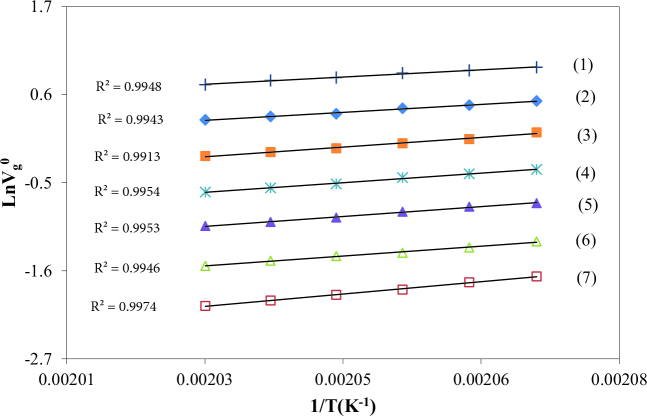
The specific retention volume diagrams, V_g_^0^, of TD (1), DD (2), UD (3), D (4), N (5), O (6) and Hp (7) on BDBB.

**Figure 7:**
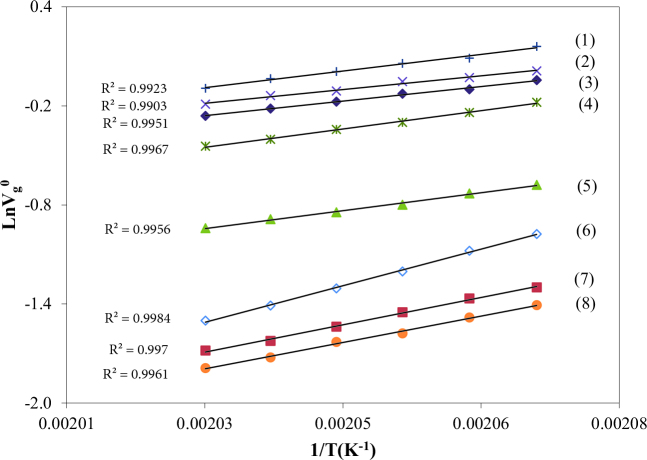
The specific retention volume diagrams, V_g_^0^, of nPB (1), iPB (2), ClB (3), EB (4), T (5), nBAc (6), iBAc (7) and EAc (8) on BDBB.

The V_g_^o^ values of the solvents on BDBB were calculated from Eq. (1). Figures 6 and 7 present the variation of LnV_g_^o^ values of Hp, O, N, D, UD, DD, TD, nBAc, iBAc, EAc, nPB, iPB, EB, ClB, and T between 483.2 and 493.2K.

LC–solvent interaction parameters, *χ* 12∞, were calculated using Eq. (2) and are shown in Table 3. 

**Table 3:** Flory-Huggins LC-solvent interaction parameters, χ12∞ , of BDBB.

Solvents	483.2 K	485.2 K	487.2 K	489.2 K	491.2 K	493.2 K
Hp	3.68	3.72	3.79	3.82	3.87	3.91
O	3.69	3.73	3.77	3.78	3.80	3.84
N	3.65	3.66	3.69	3.73	3.75	3.77
D	3.67	3.69	3.69	3.74	3.75	3.76
UD	3.65	3.69	3.70	3.72	3.73	3.74
DD	3.70	3.70	3.70	3.72	3.71	3.71
TD	3.71	3.71	3.69	3.69	3.68	3.69
nBAc	3.48	3.54	3.64	3.71	3.78	3.84
iBAc	3.58	3.62	3.67	3.73	3.78	3.81
EAc	3.13	3.18	3.24	3.27	3.33	3.37
nPB	3.05	3.08	3.08	3.09	3.10	3.12
iPB	3.04	3.05	3.04	3.06	3.05	3.07
EB	3.01	3.04	3.07	3.08	3.10	3.11
ClB	2.92	2.95	2.94	2.95	2.96	2.97
T	3.00	3.02	3.06	3.08	3.09	3.12

Standard uncertainty u is u(χ_12_^∞^)=0.01

When *χ*_12_^∞^ is less than 0.5, that signals a good solvent for LC, while a value higher than 0.5 indicates a poor solvent for LC. The values of *χ*_12_^∞^ were higher than 0.5 and indicated that none of the solvents are good for BDBB. The values of *χ*_12_^∞^ except for TD decreased with increasing column temperature, pointing to exothermic solubility. 

The weight fraction activity coefficients of the solvents at infinite dilution were calculated using Eq. (3) and the results are presented in Table 4.

**Table 4:** The weight fraction activity coefficients at infinite dilution of the solvents, Ω_1_^∞^ , of BDBB.

Solvents	483.2 K	485.2 K	487.2 K	489.2 K	491.2 K	493.2 K
Hp	140.2	147.1	157.4	163.6	171.2	178.8
O	127.7	133.4	138.3	140.0	144.0	149.6
N	113.0	114.1	117.6	122.3	125.0	126.9
D	107.3	109.0	109.9	114.6	115.9	117.7
UD	98.2	102.4	103.5	105.5	106.3	107.1
DD	97.0	97.6	96.8	98.9	97.9	97.7
TD	93.2	92.3	90.7	91.2	90.0	90.5
nBAc	89.1	95.5	104.9	112.8	121.3	128.9
iBAc	106.0	110.0	116.0	123.1	130.3	134.3
EAc	73.7	77.4	83.3	85.8	92.0	96.0
nPB	56.6	58.6	58.3	59.1	59.7	61.1
iPB	55.4	55.8	55.2	56.5	56.2	57.3
EB	56.4	58.1	59.7	60.4	62.1	62.7
ClB	42.0	43.0	42.7	43.5	43.9	44.4
T	59.1	60.6	63.0	64.2	65.1	66.9

Standard uncertainty u is u(Ω_1_^∞^)=0.1

According to Guillet, a solvent is determined as good if Ω_1_^∞^ is less than 10; on the other hand it is characterized as poor if Ω_1_^∞^ is greater than 10. The values between 5 and 10 indicate a moderate solvent for BDBB. All of the studied solvents were poor when the values of Ω_1_^∞^ were evaluated. The values of Ω_1_^∞^ decrease with increasing chain length of *n* -alkanes, as observed in this research. 

The values of partial molar heat, Δ *H* *^–^* 1∞, of mixing at infinite dilution were obtained from the slopes of the plots of Ln Ω1∞ versus 1/T using Eq. (4) between 483.2 K and 493.2 K. Results are presented in Table 5. Δ *H* *^–^* 1∞ values show that the solubility of LC in all solvents was exothermic except for TD; these results are also consistent with the values of *χ* 12∞. The values of the partial molar heat, ΔHS , of sorption of the solvents on BDBB were obtained from the slopes of the straight lines of LnVgo in the same temperature ranges using Eq. (5). The values are presented in Table 5. The values of Δ *H* *^–^* *V* determined by Eq. (8) were also compared to the literature values [29]. Table 5 shows the results for all of the researched solvents. A good consistency between the calculated and literature values of Δ *H* *^–^* *V* was detected for the solvents where the boiling temperature was close to the average of the researched column temperatures. According to Table 5, there is very good consistency between Δ *H* *^–^* *V* and Δ *H* *^–^* *VL* values for UD and DD. The boiling points of UD (boiling point 469.2 K) and DD (boiling point 489.4 K) are very close to the examined temperatures. On the other hand, there is disagreement for the Δ *H* *^–^* *V* values for the other solvents at the studied temperatures. 

**Table 5:** The values of the partial molar heat of sorption, ΔH–S (kcal mol^-1^), the partial molar heat of mixing at infinite dilution, ΔH_1_^∞^ (kcal mol^-1^), the molar heat of vaporization, ΔH–V (kcal mol^-1^), of the studied solvents, and the literature values of the molar heat of vaporization, ΔH–VL (kcal mol^-1^) [29].

Solvents	ΔH–S	ΔH–1∞	ΔH–V	ΔH–VL[29]
Hp	–17.5	–11.6	5.9	7.6
O	–14.0	–7.0	7.0	8.2
N	–14.0	–6.0	8.0	8.8
D	–13.6	–4.6	9.0	9.4
UD	–13.7	–3.8	9.9	9.9
DD	–11.3	–0.5	10.8	10.4
TD	–10.2	1.5	11.7	10.9
nBAc	–25.2	–17.8	7.4	8.6
iBAc	–18.8	–11.9	6.9	8.7
EAc	–18.1	–12.6	5.5	7.7
nPB	–11.4	–3.0	8.4	9.1
iPB	–9.4	–14.2	8.0	9.0
EB	–12.6	–5.0	7.6	8.5
ClB	–10.0	–2.4	7.6	8.7
T	–12.3	–5.8	6.5	7.9

## 4. Discussion

In this research, the synthesis and characterization of BDBB are presented, indicating enantiotropic nematic (N) and tilted smectic (SmC) phases in a broad temperature range. The phase behavior of BDBB was examined with PM, DSC, and IGC. The transition temperatures of BDBB found via IGC are compatible with those found with DSC. nBAc, iBAc, tBAc, nBAl, iBAl, and tBAl can be separated from their mixtures by IGC with the use of BDBB as a stationary phase. This research also confirms that the separation capability of BDBB is good enough for alcohol and acetate isomers in the examined temperature ranges. IGC was used for studying the thermodynamic parameters of BDBB. According to the results, the studied solvents are not good solvents for BDBB. IGC is an appropriate analysis for the examination of the thermodynamic properties of LCs.

Supplementary Materials
