# Supplementary material for: Synthesis and characterization of novel 4-benzyloxyphenyl 4-[4-(n-dodecyloxy)benzoyloxy]benzoate liquid crystal
Source: Turk J Chem. 2021 Feb 17;45(1):71–81. doi: 10.3906/kim-2007-64 (PMC7925317; doi:10.3906/kim-2007-64)
Supplement: Supplementary file 1 — Supplementary Materials [file turkjchem-45-71-sup001.pdf]

SUPPORTING INFORMATION

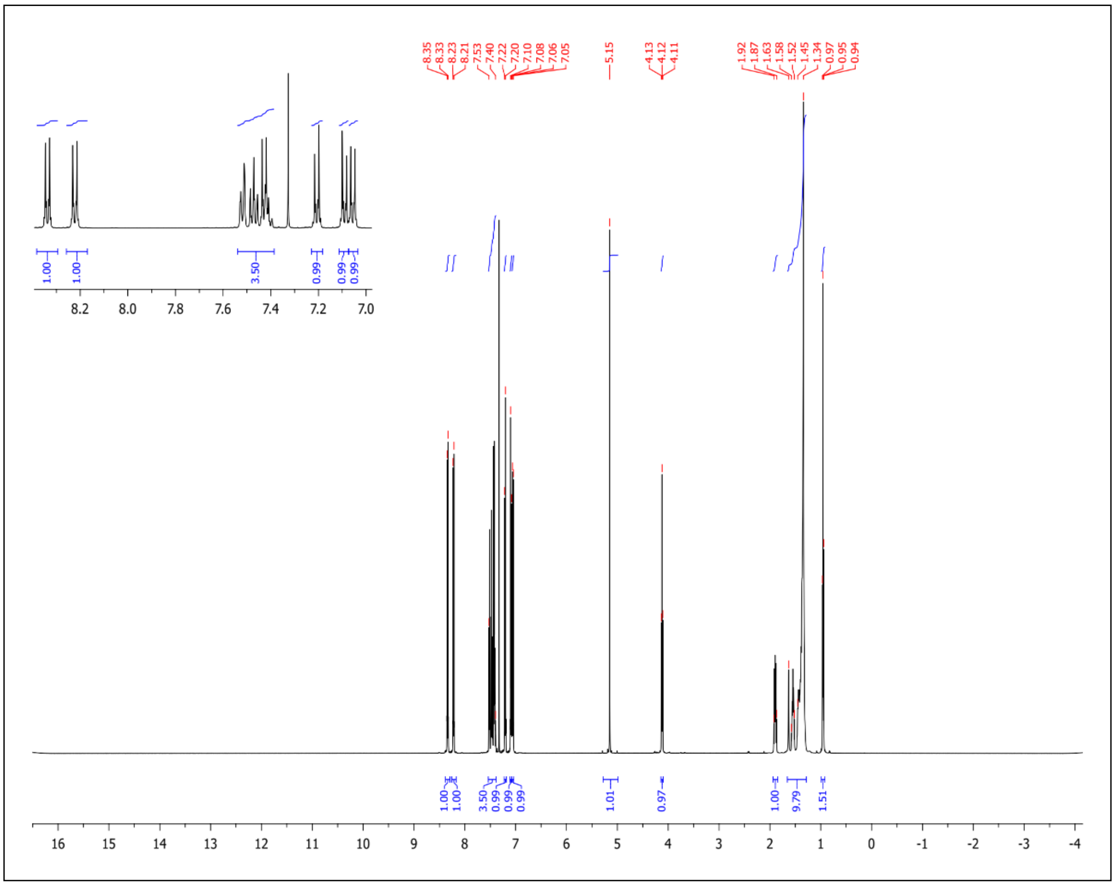

Figure S1. ^{1}H-NMR spectrum of compound BDBB (500 MHz, $CDCl_3$).

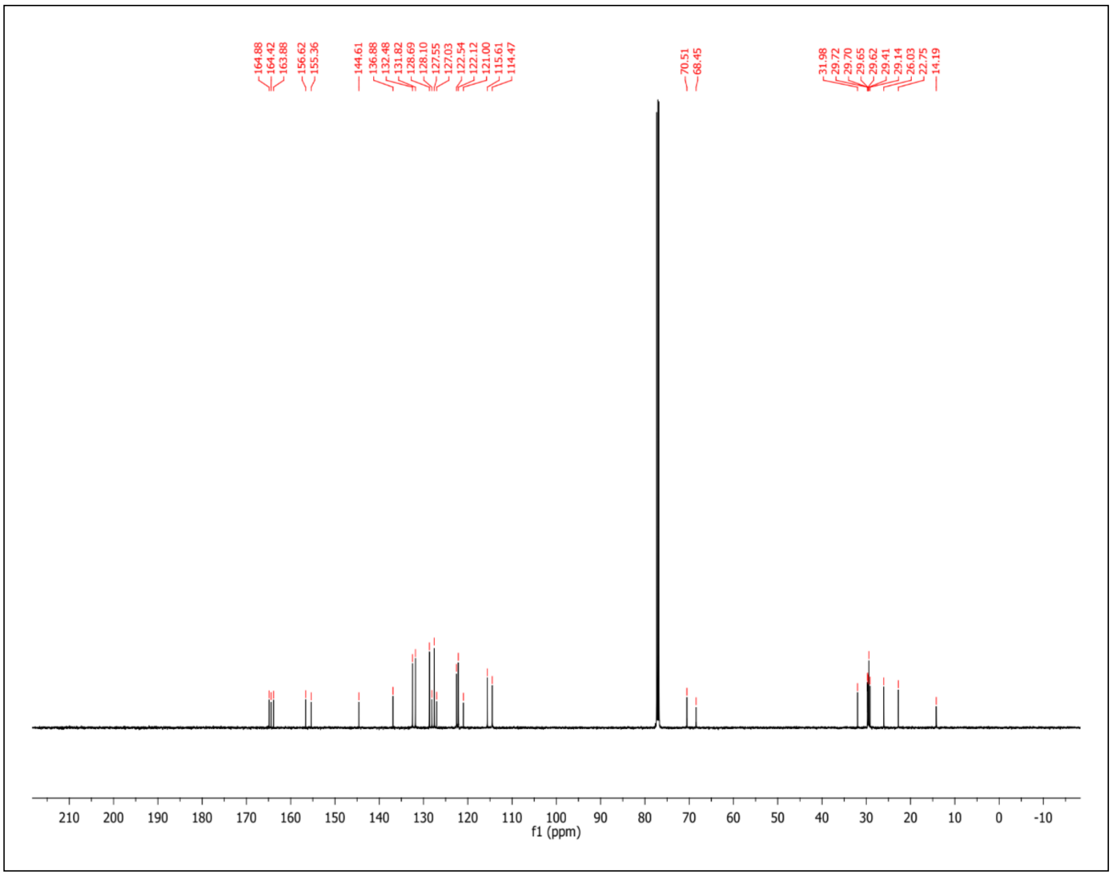

Figure S2.^{13}C-NMR spectrum of compound BDBB (125 MHz, $CDCl_3$).

Yield: 85 %; colorless crystals. **^{1}H-NMR** (500 MHz, DMSO-d_6): δ (ppm)= 8.08 (d; *J* ≈ 8.9 Hz; 2Ar-H), 8.03 (d; *J* ≈ 8.7 Hz; 2Ar-H), 7.39 (d; *J* ≈ 8.7 Hz; 2Ar-H), 7.11 (d; *J* ≈ 8.9 Hz; 2Ar-H), 4.09 (t; *J* ≈ 6.5 Hz; 2H, OCH_2), 1.77-1.72 (m; 2H, CH_2), 1.46-1.39 (m; 2H, CH_2), 1.34-1.25 (m; 16H, 8CH_2), 0.85 (t; *J* ≈ 6.9 Hz; 3H, CH_3). **^{13}C-NMR** (125 MHz, DMSO-d_6): δ (ppm)= 166.62 (COOH), 163.80 (COO), 163.36, 154.12, 128.55, 120.44 (Ar-C), 132.09, 130.83, 122.10, 114.73 (Ar-CH), 68.01 (OCH_2), 31.24, 28.96, 28.91, 28.65, 28.42, 25.35, 22.04 (CH_2), 13.89 (CH_3).

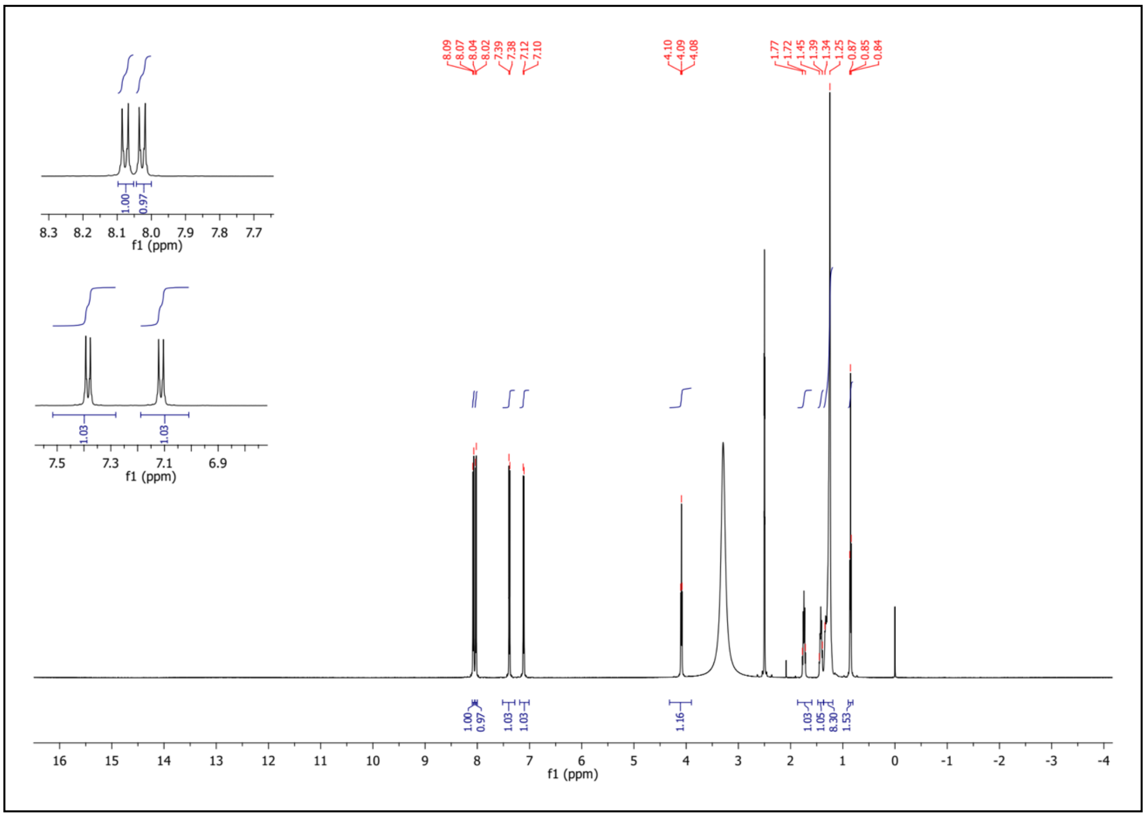

Figure S3.^{1}H-NMR spectrum of compound 4 (500 MHz, DMSO-d_6).

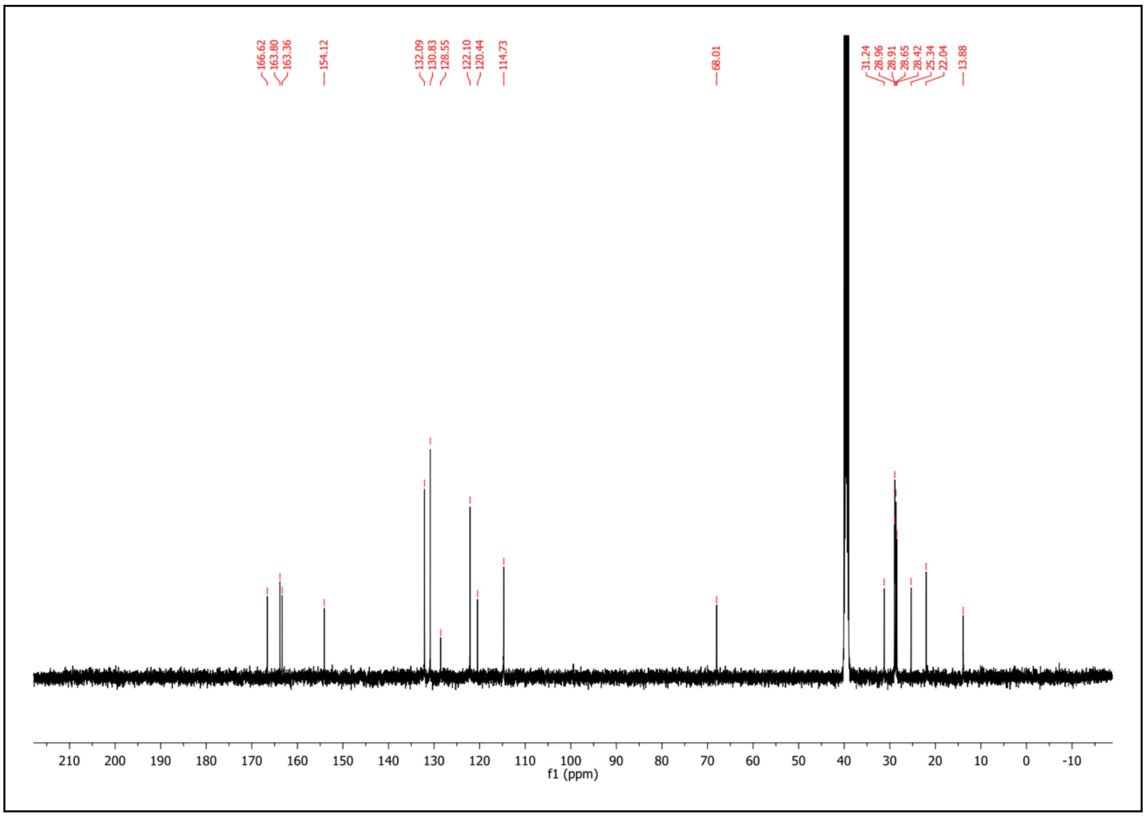

Figure S4.^{13}C-NMR spectrum of compound 4 (125 MHz, $CDCl_3$).
